# Complete genome sequence of the carotenoid producing halophilic archaeon *Haloferax volcanii* TM^T^

**DOI:** 10.1128/mra.01421-25

**Published:** 2026-07-10

**Authors:** Dana Griffiths, Crystal Young, Hussain Alattas, Ravi Tiwari, Daniel Murphy, Colin Scott, Wayne Reeve

**Affiliations:** 1 Bioplastics Innovation Hub, Food Futures Institute, Murdoch University 5673, Murdoch, Australia; 2 School of Medical, Molecular and Forensic Sciences, Murdoch University 5673, Murdoch, Australia; 3 CSIRO Environment 2220, Brisbane, Australia; Portland State University, Portland, Oregon, USA

**Keywords:** *Haloferax*, *Haloferacaceae*, complete genome, *Alexandrinus*, *Alexandrinum*, Haloararchaea, DSM27206, *volcanii*, TM^T^

## Abstract

*Haloferax volcanii* DSM27206 (formerly *Haloferax alexandrinus* TM^T^) is a halophilic red archaeon. The genome contains a circular chromosome (2,850,539 bp with a guanine-cytosine [GC] content of 66.7%) and three plasmids (5,595, 296,275, and 496,018 bp with a GC content of 64.0, 66.4, and 64.0%, respectively). National Center for Biotechnology Information Prokaryotic Genome Annotation Pipeline annotation identified 3,458 protein-encoding genes.

## ANNOUNCEMENT

*Haloferax volcanii* DSM27206 is an archaeon belonging to the Halobacteriaceae family isolated from a solar saltern in Alexandria (1, 2). *Haloferax volcanii* TM^T^ produces canthaxanthin and can accumulate silver nanoparticles (3–7). The previous published genome (ASM33673v1) has 36 contigs, and no complete genome was available (4).

A pure culture of this strain was obtained from DSMZ and grown in broth at 42°C containing (M): NaCl, 4.28; KCl, 0.027; MgSO_4_.7H_2_O, 0.081; C_6_H_5_Na_3_O_7_, 0.012; and yeast extract (10 g/L) (8). High-molecular weight DNA was obtained from a logarithmic culture using Promega’s Wizard HMW-DNA Extraction Kit using the standard protocol (9). DNA quality was checked using spectrophotometry and gel electrophoresis.

All tools were run with default parameters unless otherwise specified. Oxford Nanopore Technologies (ONT) libraries were prepared with the ONT Rapid Barcoding Kit V14 protocol and sequenced with a FLO-MIN114 flow cell (R10.4.1) using a MinION Mk1B. Guppy v3.2.6 was used for base calling (read-pass-filter quality score cutoff, seven, minimum length 1,500 bp). Long-read sequencing generated 39,915 reads for a total of 108,661,496 bp (29.78× coverage) with a read *N*_50_ value of 2,874. Short reads were obtained from National Center for Biotechnology Information (NCBI) Sequence Read Archive ERX446976 (4).

ONT long reads were assembled using Flye v2.9 with 10 iterations (10). A hybrid assembly was completed by mapping short reads to the long-read assembly using minimap2 to correct ONT-generated sequencing errors (11). The final assembly resulted in asingle circular chromosome (2,850,539 bp with a guanine-cytosine [GC] content of 66.7%) and three plasmids (5,595 bp with a GC content of 64.0%, 296,275 bp with a GC content of 66.4% and 496,018 bp with a GC content of 64.0%).

**Fig 1:**
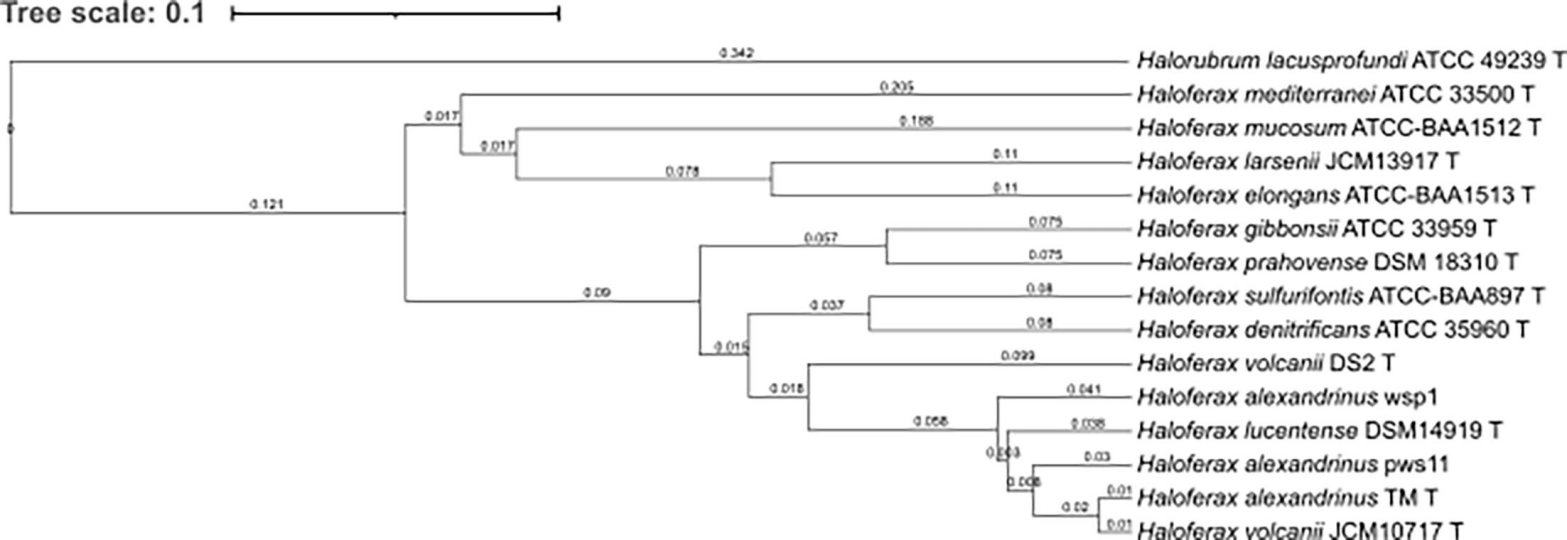
Genome-based phylogenetic relationship of species of the *Haloferax* genus (T denotes the type strain). ANIb values were calculated using JspeciesWS and DendroUPGMA, utilizing the algorithm’s similarity matrix (5, 14, 15). The generated tree was visualized in the Interactive Tree Of Life (iTOL) (16). *Halorubrum lacusprofundi* ATCC49239^T^ was used as an outgroup. *Haloferax alexandrinus* TM^T^ is the genome from this paper, and *Haloferax volcanii* JCM10717^T^ is the genome ASM33673v1. Numbers denote branch lengths.

A completeness score of 98.7% of the genome assembly was confirmed using BUSCO v5.5.0 (12). CheckM indicated a completeness of 97.54% and a contamination score of 0.38% (13). Annotation of the genome was performed using the NCBI Prokaryotic Genome Annotation Pipeline (PGAP) and summarized in Table 1. ANIb values were determined, and a phylogenetic tree (Fig. 1) was generated with JspeciesWS, DendraUPGMA, and iTOL (14–16).

**TABLE 1:** General features of the *Haloferax volcanii* genome using NCBI PGAP annotation

Feature	GenBank annotation
Total no. of genes	3,732
No. protein-coding genes	3,458
No. of rRNA operons	2
5S	2
16S	2
23S	2
No. of tRNAs	55
No. of other RNA genes	8
Locus tag prefix	V4435

This work has completed the genome for this organism, which may help to resolve the taxonomy of the strain.

## Data Availability

The complete genome sequence of *H. volcanii* TMT has been deposited in GenBank under accession numbers CP145315, CP145314, CP145313, and CP145312. The raw long sequencing reads have been deposited in GenBank under accession number SRR28058716.
